# Identification of Independent and Shared Metabolic Responses to High-Fiber and Antibiotic Treatments in Fecal Metabolome of Grow–Finish Pigs

**DOI:** 10.3390/metabo12080686

**Published:** 2022-07-26

**Authors:** Yuan-Tai Hung, Yajian Song, Qiong Hu, Richard J. Faris, Juanjuan Guo, Yiwei Ma, Milena Saqui-Salces, Pedro E. Urriola, Gerald C. Shurson, Chi Chen

**Affiliations:** 1 Department of Animal Science, University of Minnesota, 1988 Fitch Ave, Saint Paul, MN 55108, USA; hungx121@umn.edu (Y.-T.H.); msaquisa@umn.edu (M.S.-S.); urrio001@umn.edu (P.E.U.); shurs001@umn.edu (G.C.S.); 2 Department of Food Science and Nutrition, University of Minnesota, 1334 Eckles Ave, Saint Paul, MN 55108, USA; songyajian@tust.edu.cn (Y.S.); gjjfst15@163.com (J.G.); maxxx792@umn.edu (Y.M.); 3 Cargill Animal Nutrition, 10383 165th Ave NW, Elk River, MN 55330, USA; qiong_hu@cargill.com (Q.H.); richard_faris@cargill.com (R.J.F.); 4 Department of Veterinary Population Medicine, University of Minnesota, 1365 Gortner Ave, Saint Paul, MN 55108, USA

**Keywords:** fiber, wheat middling, antibiotic, bacitracin, fecal metabolome, swine

## Abstract

Feeding high-fiber (HF) coproducts to grow–finish pigs as a cost-saving practice could compromise growth performance, while the inclusion of antibiotic growth promoters (AGPs) may improve it. The hindgut is a shared site of actions between fiber and AGPs. However, whether the metabolic interactions between them could occur in the digestive tract of pigs and then become detectable in feces have not been well-examined. In this study, wheat middling (WM), a HF coproduct, and bacitracin, a peptide antibiotic (AB), were fed to 128 grow–finish pigs for 98 days following a 2 × 2 factorial design, including antibiotic-free (AF) + low fiber (LF); AF + HF; AB + LF, and AB + HF, for growth and metabolic responses. The growth performance of the pigs was compromised by HF feedings but not by AB. A metabolomic analysis of fecal samples collected on day 28 of feeding showed that WM elicited comprehensive metabolic changes, especially in amino acids, fatty acids, and their microbial metabolites, while bacitracin caused selective metabolic changes, including in secondary bile acids. Limited metabolic interactions occurred between fiber and AB treatments. Moreover, the correlations between individual fecal metabolites and growth support the usage of fecal metabolome as a source of biomarkers for monitoring and predicting the metabolic performance of grow–finish pigs.

## 1. Introduction

High-fiber (HF) coproducts from agroindustry, such as wheat middling (WM), are commonly used as low-cost feed ingredients for grow–finish pigs [1]. Even though grow–finish pigs can tolerate more fiber than nursery pigs, this practice could still decrease nutrient utilization [2] and compromise their growth performance [3]. The influences of fiber on the metabolism and growth of pigs are site-specific. In the small intestine, fiber affects the digestion and absorption of nutrients through interfering with the functions of digestive enzymes, as well as with nutrient receptors and transporters [4,5]. In the large intestine, fiber has extensive bidirectional interactions with resident microbes. On one hand, fermentable fiber is the raw material for gut microbes to produce short-chain fatty acids (SCFAs), an energy source for colonocytes and a key player in host-energy metabolism [6,7]. On the other hand, fiber can alter the composition of the microbiome and then indirectly affect the production of microbial metabolites in the hindgut and feces, including SCFAs, secondary bile acids, biogenic amines, and the degradation products of phytochemicals [8,9]. Many of these microbial metabolites have regulatory functions on metabolism, immunity, and endocrine signaling. 

Antimicrobial growth promoters (AGPs) have been used extensively in swine production to improve growth through preventing gastrointestinal morbidities, sparing nutrients used for inflammatory responses, and improving metabolic efficiency [10,11]. As antimicrobial agents, in-feed AGPs can decrease microbial populations and alter microbial compositions in the gut and feces [12,13,14]. The metabolic consequences of these changes are the altered microbial metabolism of nutrients in the large intestine. For example, pigs fed AGPs showed increased biogenic amines and skatole while they displayed decreased SCFAs in their feces compared with pigs fed control diets [12,15,16], showing that AGPs can influence microbial metabolites as a metabolic modifier. However, concerns about antibiotic resistance have led to worldwide regulations on AGP usage in animal husbandry [17]. Identifying natural growth promoters as AGP alternatives to improve feed efficiency has been a priority in research; however, an insufficient understanding of how AGPs affect the hindgut metabolism of feed ingredients has hampered efforts. Bacitracin, a natural peptide antibiotic that is not regulated by the Veterinary Feed Directive of the U.S. Food and Drug Administration [17], has been widely used as a feed additive for managing swine *Clostridium* diseases and also as a model compound to investigate the modes of action of AGPs, as well as to guide the discovery of AGP alternatives and natural growth promoters.

In commercial swine production, fiber and AGPs commonly coexist in diets. Since fiber and AGPs share gut microbiota and microbial metabolism as their targets of action in the hindgut [18,19], an interaction between fiber and AGPs in hindgut metabolism may occur. Few studies examined this topic. Antibiotic cocktails reduced oxygen consumption in the pigs fed HF diets compared with the ones fed low-fiber diets without antibiotics [20], indicating less heat production and enhanced energy utilization efficiency. Moreover, the addition of virginiamycin improved the total tract digestibility of dry matter and energy in the growing pigs fed high-fiber diets compared with the ones fed low-fiber diets [21]. Therefore, beneficial metabolic interactions could occur between antibiotics and fiber, affecting animal performance by lowering the metabolic rate and improving the efficiency of feed utilization. However, fiber- or AGPs-responsive metabolites were not reported in these studies. 

Compared with many sources of biological samples that could be used to probe fiber- and AGPs-induced metabolic changes, feces is more accessible and contains a unique metabolome derived from feed ingredients and the products of gut microbiota and the host [22]. The present study examined the metabolic interactions between bacitracin and WM in the feces of grow–finish pigs through comprehensive metabolomic analysis to reveal the metabolic signature related to growth in response to dietary intervention. 

## 2. Results

### 2.1. Effects of Fiber and Antibiotics (ABs) on Growth Performance of Grow–Finish Pigs

The four-phase feeding program showed that fiber and AB had different effects on the growth performance of grow–finish pigs (Table 1). The HF diets decreased both average daily gain (ADG) and gain efficiency (ADG/ADFI) (*p <* 0.01) compared with the LF diets, regardless of AB addition; however, they had no impact on the average daily feed intake (ADFI). In contrast, the AB treatments had no impact on ADG and gain efficiency but tended to increase (*p =* 0.08) ADFI. The pigs fed AB + LF had the highest BW among treatments. No interactions between fiber and AB (AB × Fiber) were observed in all growth parameters.

### 2.2. Targeted Analysis of Free Amino, Fatty, and Bile Acids in Fecal Samples

The effects of fiber and AB on major fecal metabolites from nutrient digestion and microbial metabolism, including free amino (AAs), free fatty, and bile acids, were evaluated by targeted quantitative analysis (Table 2). Feeding HF diets decreased (*p <* 0.05) the concentrations of all essential AAs (Met, Leu/Ile, Val, Thr, Lys, His, Phe, and Trp) and some non-essential AAs (Ala, Tyr, Ser, Glu, and Cit) in fecal samples. AB treatments did not affect the concentrations of free AAs in the feces, except for His. The fecal concentration of His was also affected by the fiber × AB interaction (*p =* 0.04), with bacitracin inclusion increasing His in LF feedings but not in HF feedings. 

Among fecal free fatty acids, the concentrations of SCFAs were not affected by either fiber or AB, while the concentrations of long-chain fatty acids (LCFAs) were selectively affected by HF but not by AB. HF diets significantly increased two monounsaturated fatty acids, i.e., oleic (C18:1; *p =* 0.02) and palmitoleic acids (C16:1; *p <* 0.01), and tended to increase linoleic acid (C18:2; *p =* 0.09) but decreased three saturated fatty acids, namely lauric (C12:0), myristic (C14:0), and pentadecanoic acids (C15:0) (*p <* 0.01, *p =* 0.01, and *p <* 0.01, respectively). No interactions between fiber and AB treatments on fecal fatty acids were observed. 

In contrast to the limited effects of AB treatments on fecal AAs and fatty acid contents, lithocholic (LCA) and hyodeoxycholic acids (HDCA), two dominant secondary bile acids in pigs, were decreased (*p <* 0.01) by AB but not by fiber. Among other minor bile acids and salts, glycochenodeoxycholic acid (GCDCA) was increased (*p =* 0.02) by AB while cholic (CA), deoxycholic (DCA), and taurocholic acids (TCA) were decreased (*p <* 0.01, *p =* 0.02, and *p =* 0.02, respectively) by the HF diet. The concentration of chenodeoxycholic acid (CDCA) was affected by the fiber × AB interaction (*p =* 0.04), with bacitracin inclusion increasing CDCA in LF-fed pigs while decreasing it in HF-fed pigs.

The correlations between these metabolites and ADG were further examined for their association with growth response (Table 2). Multiple AAs were positively correlated with ADG (*p <* 0.05), including three essential AAs (i.e., His, Lys, and Thr, with r = 0.18, 0.25, and 0.17, respectively), and non-essential AAs (i.e., Asp, Cit, Glu, Gln, and Tau, with r = 0.23, 0.20, 0.17, 0.28, and 0.19, respectively). No correlations were identified for free fatty acids, while glycocholic acid (GCA) was the only bile acid that positively correlated with ADG (r = 0.21, *p <* 0.05). 

### 2.3. Untargeted Analysis of Fiber- and Antibiotics-Elicited Changes in the Fecal Metabolome

**Figure 1:**
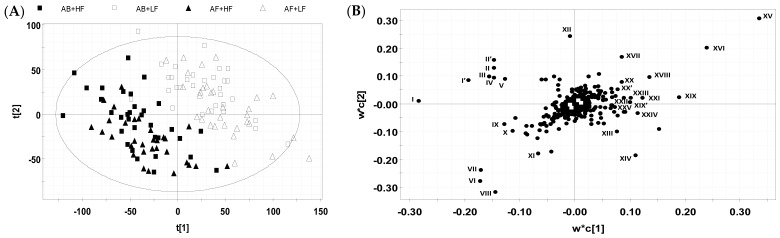
Identification of fecal metabolites altered by fiber and antibiotic in grow-finish pigs through metabolomic modeling. The LC-MS data of fecal samples from four treatment groups (AF + LF, AF + HF, AB + LF, and AB + HF) were processed by PLS-DA modelling. (**A**) The scores plot of PLS-DA model. The t[1] and t[2] are projection values of each sample in 1st and 2nd principal components of the model, respectively. (**B**) The loadings plot of PLS-DA model. The correlations of individual fecal ions with first and second components of the PLS-DA model were indicated by their respective w*c[1] and w*c[2] values. Major metabolites responsive to high fiber and antibiotic treatments are labeled and enlisted in Table 3.

Untargeted metabolomic analysis and modeling were conducted to provide a more comprehensive coverage of the small-molecule metabolites in fecal samples. In the PLS-DA model of pooled LC-MS data, the two HF treatments (AF + HF and AB + HF) were clearly separated from the two LF treatments (AF + LF and AB + LF), while the samples of two AB treatments (AB + LF and AB + HF) and two AB-free treatments (AF + LF and AF + HF) were not clearly separated (Figure 1A). This result shows that fiber caused greater changes in the fecal metabolome than AB. The major metabolites (I-XXV) contributing to the sample separation in the PLS-DA model were identified in the loadings plot (Figure 1B), and further characterized by structural and quantitative analyses (Table 3). 

**Figure 2:**
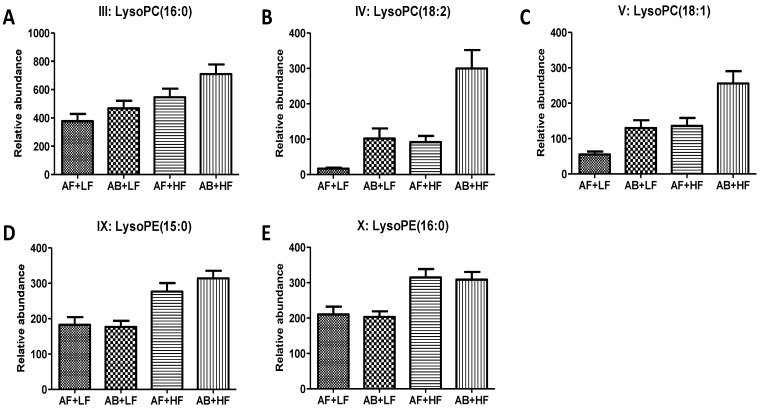
Effects of antibiotic and fiber treatments on lysophophoslipids in feces. (**A**) LysoPC(16:0), (**B**) LysoPC (18:2), (**C**) LysoPC (18:1), (**D**) LysoPE (15:0), (**E**) LysoPE (16:0). *p*-values are presented in Table 3.

**Figure 3:**
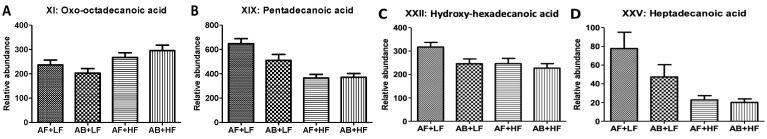
Effects of antibiotic and fiber treatments on free fatty acids in feces. (**A**) Oxo-octadecanoic acid, (**B**) Pentadecanoic acid, (**C**) Hydroxy-hexadecenoic acid, (**D**) Heptadecanoic acid. *p*-values are presented in Table 3.

Diverse lipid species were identified by untargeted metabolomics analysis as fiber- and AB-responsive metabolites (Table 3), including oleic (I), linoleic (II), and pentadecanoic acids (XIX), and LCA (XIII) and DCA (XXIII), which were also detected by the targeted analyses on amino, fatty, and bile acids (Table 2). Lysophospholipids, the digestion products of phospholipids, are a major group of fiber- and AB-responsive metabolites detected by an untargeted analysis. Among them, lysophosphatidylcholines (lysoPC), including lysoPC (16:0) (III), lysoPC (18:2) (IV), and lysoPC (18:1) (V) were increased by both AB and fiber (Figure 2A–C), while lysophosphatidylethanolamines (lysoPE), including lysoPE (15:0) (IX) and lysoPE (16:0) (X), were only increased by fiber (Figure 2D-E). Free fatty acids, as fiber- and AB-responsive metabolites, were further observed in the untargeted analysis. HF increased oleic (I) and linoleic acid (II) (Table 2 and Table 3) but decreased pentadecanoic (XIX) and heptadecanoic acid (XXV), which are two odd-chain fatty acids (Figure 3B,D). In addition, HF increased oxo-octadecanoic acid (XI), while both AB and HF decreased hydroxyhexadecanoic acid (XXII) (Figure 3A,C). 

**Figure 4:**
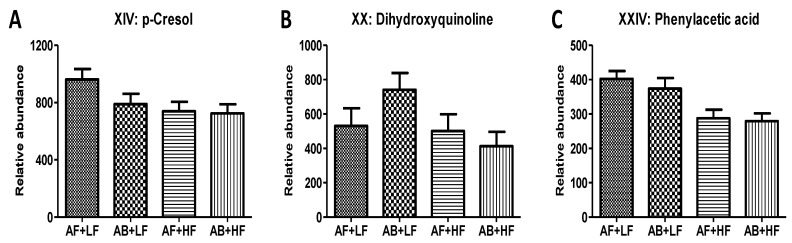
Effects of antibiotic and fiber treatments on microbial metabolites of aromatic amino acids in feces. (**A**) *p*-Cresol, (**B**) 4,6-dihydroxyquinoline, (**C**) phenylacetic acid. *p*-values are presented in Table 3.

**Figure 5:**
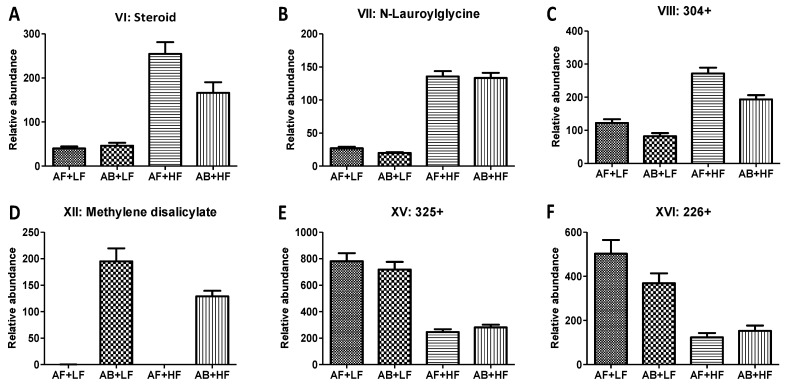
Potential exposure metabolite markers of antibiotic and fiber treatments in feces. (**A**) Steroid, (**B**) *N*-lauroylglycine, (**C**) 304+, (**D**) Methylene disalicylate, (**E**) 325+, (**F**) 226+. *p*-values of treatments are presented in Table 3.

The untargeted analysis further confirmed the negative effects of HF treatments on free AAs in feces (Table 2 and Table 3). Interestingly, *p*-cresol (XIV), dihydroxyquinoline (XX), and phenylacetic acid (XXIV), which are the respective microbial metabolites of three aromatic AAs, i.e., Tyr, Trp, and Phe, were also decreased by HF treatments (Figure 4A–C). Other fecal metabolites that were only affected by either fiber or AB were identified (Figure 5). Among them, methylene disalicylate (XII), an organic acid that forms a complex with bacitracin for solubility, was only detected in the fecal samples of pigs fed AB (Figure 5D). HF treatments increased *N*-lauroylglycine (VII) and two undefined fecal metabolites, namely VI (a cholesterol metabolite) and VIII (Figure 5A–C), while they decreased metabolites XV and XVI (Figure 5E,F). The correlations between metabolite markers and growth were also identified (Table 3). Metabolite XV and 2-aminooctanoic acid (XXI) were positively correlated with ADG, with r = 0.18 and 0.20, respectively, while *N*-lauroylglycine (VII) and unknown metabolite (VIII) were negatively correlated with ADG, with r = −0.31 and −0.25, respectively. 

## 3. Discussion

The metabolomic analysis in this study showed prominent changes from WM treatments and subtle changes for bacitracin exposure in the metabolite content of swine feces. The causes and significances of these changes are discussed largely based on the biochemical properties of responsive metabolites, as well as the high-fiber nature of WM and antibiotic nature of bacitracin. Furthermore, individual metabolic changes that were independent of the fiber function of WM or the antibiotic function of bacitracin are also discussed.

### 3.1. Causes and Significances of WM Effects on Growth and Fecal Metabolome of Grow–Finish Pigs 

The fiber and other bioactive contents in WM have diverse metabolic functions, which were reflected by the HF-elicited changes in amino acid and lipid metabolites in feces, as well as their correlations with pig growth in this study.

#### 3.1.1. Effects of WM on Growth Performanc

WM inclusion in the HF treatments increased neutral detergent fiber (NDF) content from 37% to 87% relative to NDF content in LF treatments from phase 1 to phase 4. Because increased NDF intake linearly reduces the digestibility of nutrients and energy in pigs [23], decreased growth performance in pigs fed HF diets was not unexpected in the current study. In fact, our results are in agreement with previous studies, in which increased inclusion of WM decreased the ADG and gain efficiency but not the ADFI of pigs [3,24].

#### 3.1.2. Effects of WM on Fecal Amino Acid Metabolites

Decreases in all free essential AAs and most nonessential AAs in the feces are the prominent changes in the fecal metabolome caused by the HF treatments. This observation reflects the known negative effects of dietary NDF on AA digestibility in pigs [25]. Among essential AAs, the decreases in His, Lys, and Thr in feces were correlated with the decreases in ADG, supporting their important nutritional value in growth-related bioactivities. Compared with essential AAs, which only come from the diet, nonessential AAs in feces could also be originated from intestinal and microbial metabolism. Two of them, Cit and Gln, had their fecal concentrations correlated with ADG. Interestingly, endogenous Cit is mainly produced by enterocytes through Gln metabolism [26]. Gln is a major source of energy for enterocytes and has roles in the proliferation, barrier function, and stress responses of pig intestinal cells [27], while circulating Cit has been suggested as an indicator of the intestinal health and function of pigs [27,28]. Previous studies have shown that feeding wheat bran, which is the source of fiber in WM, led to colonic mucosal cell hyperplasia in rats [29], and HF diets also increased the intestinal cell turnover rate in pigs [30]. Therefore, the observed decreases in fecal Gln and Cit might reflect a WM-fiber-elicited elevation of Gln catabolism by the intestinal cells, yielding less available Gln and Cit for fecal excretion.

Besides AAs, microbial metabolites of aromatic AAs, including *p*-cresol (XIV) from Tyr, dihydroxyquinoline (XX) from Trp, and phenylacetic acid (XXIV) from Phe, were also decreased by WM. This observation could be simply explained as the consequences of decreased supplies of aromatic AAs for microbial metabolism, as shown by the reduced concentrations of Tyr, Trp, and Phe in feces after WM treatments. Nevertheless, another potential contributing mechanism is the competitive inhibition of bacterial enzymes responsible for producing these metabolites from aromatic AAs, since phenolics, especially ferulic acid, in WM undergo the same reactions in their microbial metabolism as aromatic AAs. Our recent study also showed that the decreases in microbial metabolites of aromatic AAs, including *p*-cresol and indoxyl sulfate and phenylacetylglycine, occurred in the mice fed wheat bran [31]. 

#### 3.1.3. Effects of WM on Fecal Lipids

Multiple free fatty acids and fatty acid metabolites were affected by WM. However, SCFAs (C2–C5) were not among them. Considering that the fiber in WM mainly comprises insoluble fiber fractions and partially fermentable arabinoxylans, this observation is not surprising [32]. Furthermore, it is worth considering that fecal SCFAs may not be an entirely accurate reflection of the intestine, as they are not static and continuously metabolized for multiple functions. Instead, WM decreased pentadecanoic, heptadecanoic, and 2-aminooctanoic acids, while it increased oleic and oxo-octadecanoic acid and *N*-lauroylglycine. As two odd-chain fatty acids from microbial metabolism, pentadecanoic (C15:0) and heptadecanoic acids (C17:0) were produced by either the biosynthesis starting with propionyl-CoA or the α-oxidation reactions that remove one carbon from respective even-chain fatty acids [33]. Because fecal propionic acid was not affected in the WM samples, the inhibition of microbial α-oxidation reactions is a plausible cause underlying the decreases in odd-chain fatty acids. This explanation can be further consolidated if the structure of hydroxy-hexadecenoic acid (XXII), which was also decreased by WM treatments, could be confirmed as 2-hydroxy-hexadecanoic acid, which is the direct precursor of pentadecanoic acid in the α-oxidation reaction. Similarly, the increases in oleic and oxo-octadecanoic acid could also be attributed to microbial metabolism, since 10-oxo-octadecanoic acid, a plausible structure for oxo-octadecanoic acid (XI), is an intermediate metabolite in the microbial conversion of linoleic to oleic acid by gut lactic acid bacteria. Interestingly, 10-oxo-octadecanoic acid has been shown to increase energy expenditure and restrain weight gain in mice through regulating host-energy metabolism [34]. *N*-Lauroylglycine and 2-aminooctanoic acid, two fatty acid metabolites, had negative and positive correlations with ADG, respectively. As a lipoamino acid in the cell membrane of various bacteria, *N*-lauroylglycine in mouse feces has been positively correlated with *Erysipelotrichaceae* [35], which is a family of Gram-positive bacteria with the genomic characteristic of a polysaccharide utilization function [36]. As for 2-aminooctanoic acid, it has been detected in the serum of pigs as a marker of ractopamine—a growth promoter [37]. Further studies are needed to determine the chemical natures and functions of these fatty acid metabolites in growing pigs. 

HF treatments also increased two lysophosphatidylethanolamines (LysoPEs) and three lysophosphatidylcholines (LysoPCs) in feces. These lysoPEs and lysoPCs are produced by hydrolyzing the sn-2 fatty acids from the respective PEs and PCs through phospholipase A2, a ubiquitous enzyme in pancreatic juice and intestinal and bacterial cells. The sources of PEs and PCs for producing fecal lysophospholipids include bile, intestinal epithelial cell shedding, bacteria, and mucin [38,39], which can all be affected by dietary fiber in pigs [30,40,41,42]. Considering that the membrane lipids of bacteria are rich in PEs but deficient in PCs [43], the lipids from bacterial metabolism could contribute to the HF-elicited increases in fecal lysoPEs, while the increases in fecal lysoPCs were more likely for unabsorbed dietary lipids, mucin secretion, and cell shedding.

### 3.2. Causes and Significances of Antibiotic Effects on Fecal Metabolome of Grow–Finish Pigs

The addition of bacitracin did not change weight gain, though it tended to improve feed intake in the current study. This result is not surprising, as growth performance enhancement by AGPs is more commonly observed in nursery pigs [10,44], and the grow–finish pigs in this study were raised through good barn hygiene. Nevertheless, the metabolic influences of bacitracin were still detected through fecal metabolomic analysis. 

#### 3.2.1. Methylene Disalicylate as an Exposure Marker

As a natural polypeptide antimicrobial produced by *Bacillus licheniformis*, bacitracin can be effectively degraded by microbial proteolysis to AAs. This property is generally considered as an advantage of bacitracin over other chemical antibiotics on environmental impacts and might also explain the absence of bacitracin as a fecal metabolite marker of AB treatments in this study. Instead, methylene disalicylate, the stabilizer in the bacitracin product [45], was identified as an exposure marker. This observation indicates the stability of methylene disalicylate. However, our literature search showed little published information on its disposition and bioactivities in the environment [46], which could be a topic for further investigation.

#### 3.2.2. Changes in Histidine and Bile Acids

In contrast to the extensive effects of HF on AAs and fatty acids, bacitracin treatments only increased His, mainly under LF cotreatment. As an essential AA that can be synthesized by gut microbes, this observation suggested that bacitracin might promote the microbial production of His. Moreover, different from the reductive effects of HF on minor bile acids, i.e., CA, DCA, and GCDCA, bacitracin decreased HDCA and LCA, the two most abundant secondary bile acids in feces. *Clostridia* bacteria, such as *Clostridium scindens,* are active in converting primary BA to secondary BA through their 7α-dehydroxylase [47,48]. Our recent study has shown that fecal HDCA, LCA, and HDCA were positively correlated with the abundance of *Clostridia* bacteria in pigs [49]. As a polypeptide antibiotic targeting Gram-positive bacteria, bacitracin has been used to treat clostridial diarrhea in pigs [50]. Therefore, it is plausible that feeding bacitracin decreases the *Clostridia* bacterial class, which is involved in production of LCA and HDCA. 

#### 3.2.3. Changes in Other Fecal Metabolites

In contrast to the decreases in major bile acids, bacitracin, together with HF, increased lysoPCs in feces. This effect might not be simply attributed to the modulatory effects of bacitracin on gut microbes, since PCs are not the major compositional lipids in bacteria [43]. Interestingly, mastoparans, which are the same as bacitracin, and belong to the family of cationic polypeptide antimicrobials, have been shown to stimulate phospholipase activity [51]. Bacitracin’s effects on phospholipase activity and its influence on other fecal metabolites (VI, VIII, and XXII) could be explored further in future studies.

### 3.3. Implications and Limitations of Observed Metabolic Changes from Feeding WM and Bacitracin

Overall, the metabolic responses to WM, a common NDF-rich feed ingredient, and bacitracin, a natural cationic antimicrobial peptide, could reflect many metabolic events in contemporary feeding practices using NDF-rich ingredients and antimicrobial peptide AGPs. Our results showed their effects were additive, with limited synergistic or antagonistic interactions. It is likely that different metabolic responses and interactions in the fecal metabolome could occur when feeding HF ingredients with different solubilities and fermentabilities, and other AGPs with different mechanisms of action. One trial is insufficient to justify the general responses and more research efforts are needed to explore the values of fecal metabolites as the markers of pig growth, health, and wellbeing under different feeding practices. 

## 4. Materials and Methods

### 4.1. Chemicals and Reagents

We enlist the sources of the chemicals and reagents used in the chemical and LC-MS analysis and the structural confirmation and in the supplementary material (Table S1).

### 4.2. Animals, Experimental Design, and Housing

Handling and experimental procedures were approved by Cargill Animal Nutrition Committee on Animal Use for Research and Scientific Purposes, following guidelines in Directive 2010/63/EU. 

We selected a total of 128 barrows and gilts from 192 pigs ((Yorkshire × Landrace) × Duroc breed from Genesus Inc., Oakville, MB, Canada) that previously received zinc oxide (ZnO) at either 150 or 2500 ppm with and without carbadox (55 ppm; Phibro Animal Health, Fairfield, NJ, USA) in a nursery trial [52]. We removed high doses of ZnO from diets three weeks before the start of the experiment and we provided antibiotics throughout nursery (feeding carbadox) and grow–finish phases (feeding bacitracin at 25 ppm). We allocated pigs that were provided antibiotics to antibiotic treatments in the current experiment. In the current study, we housed 128 pigs (BW = 24.65 ± 4.10 kg, 64 d age) individually. We assigned them to blocks based on weight, sex, house, and nursery ZnO treatment and allocated 4 dietary treatments in a 2 × 2 factorial arrangement with two dietary fiber levels (low fiber, LF or high fiber, HF) and two levels of bacitracin (0 or 25 ppm). This resulted in 4 dietary treatments: antibiotic-free and low fiber (AF + LF), antibiotic and low fiber (AB + LF), antibiotic-free and high fiber (AF + HF), and antibiotic and high fiber (AB + HF). We formulated the LF and HF diets to be 10% NDF and 16% NDF on average, respectively, in a 4-phase feeding program (Phase 1: day 0 to 21, Phase 2: day 21 to 42, Phase 3: day 42 to 70, and Phase 4: day 70 to 98). We formulated all diets to meet or exceed the nutritional requirements of grow–finish pigs suggested by Cargill Nutrition System (Table S2). We provided pigs with ad libitum access to their assigned experimental diets and water during 98-day study. We housed pigs in climate-controlled rooms with 32 pens per room. Each pen (1.07 m × 1.98 m) consisted of concrete slatted flooring, with a dry feeder and a nipple drinker.

### 4.3. Data and Sample Collection

We weighed pigs individually on days 0, 21, 42, 70, and 98 in grow–finish phases to calculate average daily gain (ADG). We determined average daily feed intake (ADFI) from feed delivery data. We calculated gain efficiency as the ratio between ADG and ADGI (ADG/ADFI), which is inverse to feed conversion ratio (FCR). In addition, we collected fecal samples of all pigs (*n* = 32 pigs/treatment) from rectum on day 28 of feeding, snap-froze them, and stored them at −80 °C for metabolomic analysis. We selected day 28 instead of 98 for fecal collection because we observed rapid changes in fecal metabolites after 2 weeks of feeding high-resistant starch to growing pigs [22]. We performed further examinations regarding correlations between individual fecal metabolites (on day 28) and growth (on day 98) to determine whether the fecal metabolite markers detected in the early phase of feeding could function as potential prediction markers for the performance at the end of feeding.

### 4.4. Metabolomics

The LC-MS-based metabolomic analysis comprised sample preparation, chemical derivatization, LC-MS analysis, data deconvolution and processing, multivariate data analysis (MDA), and marker characterization and quantification, as previously described [53].

#### 4.4.1. Sample Preparation

We prepared fecal samples by mixing with 50% aqueous acetonitrile in 1:10 (*w*/*v*) ratio and then centrifuging at 18,000× *g* for 10 min to obtain fecal extract supernatants. 

#### 4.4.2. Chemical Derivatization

Briefly, we mixed 5 μL of sample or standard with 5 µL of 100 μmol/L *p*-chlorophenylalanine (internal standard), 50 µL of 10 mmol/L sodium carbonate, and 100 μL of DC solution (3 mg/mL in acetone). We incubated the mixture at 25 °C for 15 min and centrifuged at 18,000× *g* for 10 min. Then, we transferred the supernatant into a sample vial for LC-MS analysis. We derivatized samples with HQ prior to the LC-MS analysis to detect carboxylic acids, aldehydes, and ketones [54]. Briefly, we added 2 μL of sample into 100 μL of freshly prepared acetonitrile solution containing 1 mmol/L DPDS, 1 mmol/L triphenylphosphine, and 1 mmol/L HQ. We incubated the reaction mixture at 60 °C for 30 min, chilled on ice, and then mixed with 100 μL of ice-cold deionized water. After we centrifuged at 18,000× *g* for 10 min, we transferred the supernatant into a HPLC vial for LC-MS analysis.

#### 4.4.3. Conditions of LC-MS Analysis

We injected a 5 μL aliquot into an ultraperformance liquid chromatography quadrupole time-of-flight mass spectrometry (UPLC-QTOFMS) system (Waters, Milford, MA, USA) and separated by a BEH C18 column (Waters) with a gradient of mobile phase ranging from water to 95% aqueous acetonitrile containing 0.1% formic acid over a 10 min run. We maintained capillary and cone voltage for electrospray ionization at 3 kV and 30 V for positive mode detection, respectively. We set source and desolvation temperatures at 120 °C and 350 °C, respectively. We used nitrogen as both cone (50 L/h) and desolvation gas (600 L/h), and we used argon as collision gas. For accurate mass measurement, we calibrated the mass spectrometer with sodium formate solution (range *m*/*z* 50–1000) and monitored by the intermittent injection of the lock mass leucine-enkephalin ([M + H]^+^ = 556.2771 *m*/*z*) in real time. We acquired mass chromatograms and mass spectral data and processed by MassLynx^TM^ software version 4.2 (Waters) in centroided format. We obtained additional structural information by tandem MS (MS/MS) fragmentation with collision energies ranging from 15 to 40 eV. 

#### 4.4.4. Data Analysis and Visualization

After data acquisition in the UPLC-QTOFMS system, we deconvoluted chromatographic and spectral data of samples by MarkerLynx^TM^ software (Waters) to generate a multivariate data matrix containing information on sample identity, ion identity (retention time and *m*/*z*) and ion abundance. We calculated the abundance of each ion by normalizing the single ion counts versus the total ion counts in the entire chromatogram. Then, we exported the data matrix into SIMCA-P+^TM^ version 14.0 software (Umetrics, Kinnelon, NJ, USA) and transformed by *Pareto* scaling. We used supervised partial-least-squares-discriminant analysis (PLS-DA) to model the fecal samples. We identified metabolite markers by analyzing ions contributing to sample separation in PLS-DA models. 

#### 4.4.5. Characterization, Quantification, and Pathway Analysis of Metabolite Markers

We determined the chemical identities of metabolite markers by accurate mass measurement, using elemental composition analysis, searching the Human Metabolome Database (HMDB) and METLIN database [55], as well as performing MS/MS fragmentations and comparisons with authentic standards if available. We determined individual metabolite concentrations by calculating the ratio between the peak area of each metabolite and the peak area of the internal standard, and fitting with a standard curve using QuanLynx^TM^ software version 4.2 (Waters). 

### 4.5. Statistical Analysis

We analyzed growth performance data using repeated measures model with individual pigs as experimental units via the PROC MIXED procedure in SAS (SAS Inst. Inc., Cary, NC, USA). In the model for data analysis, we included the fixed effects of antibiotic, fiber, time, and their 2-way and 3-way interactions, and the random effects of weight, room, and sex. We analyzed targeted metabolites data using PROC MIXED procedure in SAS; however, we removed the time effect in the model. We performed multiple comparisons among treatments using PDIFF and adjusted by Tukey for multiple comparisons of means. We carried out Pearson correlation analysis to determine the relationship between treatment-responsive metabolites and ADG. We reported all mean values as least squares mean. We considered significant differences between multiple comparisons if *p <* 0.05, and between statistical trends if 0.05 *≤ p <* 0.1.

## 5. Conclusions

In the current study, feeding HF diets with up to 55% of WM decreased the growth performance of grow–finish pigs, while bacitracin had insignificant effects. The WM-based HF feeding elicited more comprehensive changes in the fecal metabolome, especially AAs, FAs, and their microbial metabolites. In contrast, bacitracin elicited more selective metabolic changes, such as secondary bile acids, and had limited shared targets with HF in fecal metabolome. Overall, the co-administration of antibiotics and fiber did not lead to extensive metabolic interactions. The identification of the correlations between individual fecal metabolites and the growth of grow–finish pigs supports the usage of fecal metabolome as the source of biomarkers for monitoring and investigating the performance of pigs under dietary modifications and health challenges.

## Figures and Tables

**Table 1:** Growth performance of pigs fed diets containing fiber and bacitracin.

	Treatment Group ^1^				SE ^2^	*p*-Values ^3^		
	AF + LF	AB + LF	AF + HF	AB + HF		AB ^4^	Fiber	AB × Fiber ^4^
**Body weight, kg**						0.16	<0.01	0.49
Initial	25.04	24.45	25.50	24.34	2.02			
Phase 1 (d0–21)	44.15	41.80	43.13	41.00	2.03			
Phase 2 (d21–42)	64.90	63.94	63.43	61.98	2.03			
Phase 3 (d42–70)	93.81	94.15	90.15	90.50	2.03			
Phase 4 (d70–98)	122.75	124.72	118.41	118.41	2.03			
**Average daily feed intake (ADFI), kg**						0.08	0.81	0.65
Phase 1 (d0–21)	1.66	1.62	1.63	1.59	0.2			
Phase 2 (d21–42)	2.28	2.38	2.29	2.30	0.2			
Phase 3 (d42–70)	2.82	2.98	2.84	2.97	0.2			
Phase 4 (d70–98)	3.33	3.36	3.46	3.44	0.2			
Overall	2.52	2.58	2.54	2.58	0.19			
**Average daily gain (ADG), kg**						0.16	<0.01	0.88
Phase 1 (d0–21)	0.89	0.81	0.83	0.81	0.05			
Phase 2 (d21–42)	1.01	1.06	0.97	0.99	0.05			
Phase 3 (d42–70)	1.03	1.08	0.96	1.01	0.05			
Phase 4 (d70–98)	1.06	1.09	1.02	1.03	0.05			
Overall	1.00	1.01	0.94	0.96	0.05			
**Gain efficiency (ADG/ADFI)**						0.70	<0.01	0.53
Phase 1 (d0–21)	0.53	0.51	0.51	0.50	0.008			
Phase 2 (d21–42)	0.44	0.45	0.43	0.44	0.008			
Phase 3 (d42–70)	0.37	0.37	0.34	0.35	0.008			
Phase 4 (d70–98)	0.32	0.33	0.30	0.30	0.008			
Overall	0.42	0.41	0.39	0.40	0.006			

^1^ AF + LF = Antibiotic-free and low-fiber diet; AB + LF = antibiotic and low-fiber diet; AF + HF = antibiotic-free and high-fiber diet; AB + HF = antibiotic and high-fiber diet. ^2^ SE = Pooled standard error of means, *n* = 32 pigs/treatment. ^3^ *p*-values were obtained from type 3 tests of fixed effects in overall model of mixed procedure. ^4^ AB = Bacitracin; AB × fiber = interaction effect between bacitracin and Fiber.

**Table 2:** Concentrations of free amino, free fatty, and bile acids in feces.

	Treatment Group ^1^				SE ^2^	*p*-Values ^3^			ADG (r Value) ^5^
	AF + LF	AB + LF	AF + HF	AB + HF		AB ^4^	Fiber	AB × Fiber ^4^	
**Amino acids, µg/g**									
Alanine	72.28	72.52	49.39	50.61	5.17	0.89	<0.01	0.92	0.15
Arginine	0.94	1.84	1.23	1.24	0.35	0.19	0.66	0.21	0.02
Aspartic acid	317	403	275	312	49	0.14	0.11	0.55	**0.23**
Citrulline	19.14	24.27	14.01	16.14	2.90	0.15	0.01	0.56	**0.20**
Glutamic acid	835	898	724	816	75	0.27	0.17	0.84	**0.17**
Glutamine	0.71	0.74	0.23	0.12	0.14	0.75	<0.01	0.57	**0.28**
Glycine	61.38	69.35	46.55	59.73	13.6	0.44	0.37	0.85	−0.04
Histidine	2.00	3.36	1.94	2.09	0.29	0.01	0.02	0.04	**0.18**
Leucine/Isoleucine	21.97	19.13	11.55	12.36	2.26	0.65	<0.01	0.42	0.08
Lysine	193	214	143	159	15	0.21	<0.01	0.87	**0.25**
Methionine	3.56	3.54	2.49	2.15	0.35	0.60	<0.01	0.65	0.16
Ornithine	6.91	5.61	2.94	6.64	1.98	0.54	0.46	0.21	0.05
Phenylalanine	14.81	15.16	8.55	10.45	2.11	0.59	0.01	0.71	0.07
Proline	36.34	38.03	31.42	36.49	4.43	0.44	0.46	0.70	0.05
Serine	10.86	12	7.70	8.46	1.56	0.51	0.02	0.90	0.12
Taurine	0.55	1.05	0.56	0.52	0.24	0.27	0.21	0.20	**0.19**
Threonine	11.60	13.71	7.83	8.55	1.10	0.20	<0.01	0.53	**0.17**
Tryptophan	1.02	1.15	0.84	0.79	0.14	0.76	0.02	0.41	0.06
Tyrosine	20.03	24.45	11.23	16.09	3.35	0.17	0.01	0.95	0.06
Valine	38.53	33.84	24.35	25.06	3.68	0.59	<0.01	0.46	0.07
γ-Aminobutyric acid	0.78	1.17	1.04	0.75	0.27	0.83	0.77	0.19	0.15
**Fatty acids, mg/g**									
Acetic acid	10.23	9.48	13.36	13.70	2.46	0.14	0.93	0.82	−0.01
Propionic acid	6.21	6.29	8.72	8.46	1.74	0.18	0.96	0.92	−0.02
Butyric acid	5.37	5.43	7.25	7.15	1.40	0.20	0.99	0.95	−0.02
Isovaleric acid	3.56	4.29	3.60	4.21	0.80	0.41	0.98	0.95	−0.02
C6:0	0.06	0.09	0.09	0.07	0.02	0.85	0.96	0.28	0.01
C8:0	0.001	0.001	0.001	0.001	0.0002	0.69	0.26	0.90	0.06
C12:0	0.004	0.003	0.002	0.002	0.0003	0.27	<0.01	0.18	−0.11
C14:0	0.08	0.07	0.06	0.06	0.01	0.56	<0.01	0.14	−0.15
C15:0	0.18	0.15	0.11	0.11	0.01	0.13	<0.01	0.07	−0.09
C16:0	2.22	2.21	2.12	2.45	0.19	0.39	0.73	0.35	−0.04
C16:1	0.007	0.007	0.010	0.011	0.001	0.24	<0.01	0.71	−0.02
C18:0	2.22	2.09	1.98	2.39	0.19	0.47	0.88	0.16	−0.03
C18:1	1.71	2.02	2.15	2.26	0.15	0.14	0.02	0.48	−0.02
C18:2	2.17	2.93	2.95	2.92	0.23	0.10	0.09	0.08	0.09
**Bile acids, µg/g ^6^**									
CA	0.40	0.37	0.21	0.20	0.05	0.70	<0.01	0.89	0.04
CDCA	0.14	0.34	0.26	0.20	0.07	0.26	0.92	0.04	0.13
DCA	0.66	0.53	0.42	0.32	0.11	0.22	0.02	0.89	0.18
HDCA	671	571	720	383	70	<0.01	0.32	0.09	0.10
LCA	549	385	509	310	58	<0.01	0.32	0.76	−0.09
GCA	0.17	0.21	0.09	0.15	0.04	0.21	0.09	0.75	**0.21**
GCDCA	0.04	0.2	0.10	0.14	0.04	0.02	0.92	0.14	−0.01
TCA	0.15	0.15	0.10	0.02	0.04	0.29	0.02	0.23	0.05

^1^ AF + LF = Antibiotic-free and low-fiber diet; AB + LF = antibiotic and low-fiber diet; AF + HF = antibiotic-free and high-fiber diet; AB + HF = antibiotic and high-fiber diet. ^2^ SE = Pooled standard error of means, *n* = 32 pigs/treatment. ^3^ *p*-values were obtained from type 3 tests of fixed effects in overall model of mixed procedure. ^4^ AB = Bacitracin; AB × fiber = interaction effect between bacitracin and fiber. ^5^ Pearson correlation coefficient between metabolite and ADG. The numbers in bold mean *p* < 0.05. ^6^ CA = Cholic acid; CDCA = chenodeoxycholic acid; DCA = deoxycholic acid; HDCA = hyodeoxycholic acid; LCA = lithocholic acid; GCA = glycocholic acid; GCDCA = glycochenodeoxycholic acid; TCA = taurocholic acid.

**Table 3:** Fecal metabolites affected by high-fiber and antibiotic treatments.

ID	Identity (Derivative) ^2^	*m*/*z* of Charged Ion	Formula of Original Molecule	Database	*p*-Values			ADG (r Value) ^1^
					AB ^3^	Fiber ^3^	AB × Fiber	
**I**	Oleic acid Oleic acid (HQ)	281.2478− 424.3321+	C_18_H_34_O_2_	HMDB00207	0.04 (↑)	<0.01 (↑)	0.42	−0.01
**II** **II’**	Linoleic acid Linoleic acid (HQ)	279.2322− 422.3164+	C_18_H_32_O_2_	HMDB00673	0.08	0.01 (↑)	0.26	0.10
**III**	LysoPC(16:0)	496.3418+	C_24_H_50_NO_7_P	HMDB10382	0.03 (↑)	<0.01 (↑)	0.53	−0.03
**IV**	LysoPC(18:2)	520.3417+	C_26_H_50_NO_7_P	HMDB10386	<0.01 (↑)	<0.01 (↑)	0.05	−0.06
**V**	LysoPC(18:1)	522.3568+	C_26_H_52_NO_7_P	HMDB10385	<0.01 (↑)	<0.01 (↑)	0.32	−0.05
**VI**	a steroid ^3^	379.2965+	C_27_H_38_O	HMDB60512	0.03 (↓)	<0.01 (↑)	0.01	−0.13
**VII**	*N*-Lauroylglycine	258.2066+	C_14_H_27_NO_3_	HMDB13272	0.44	<0.01 (↑)	0.70	**−0.31**
**VIII**	ND ^4^	304.3005+	ND		<0.01 (↓)	<0.01 (↑)	0.16	**−0.25**
**IX**	LysoPE(15:0)	440.2784	C_20_H_42_NO_7_P	HMDB11502	0.43	<0.01 (↑)	0.35	−0.11
**X**	LysoPE(16:0)	454.294+	C_21_H_44_NO_7_P	HMDB11503	0.58	<0.01 (↑)	0.76	−0.08
**XI**	Oxo-octadecanoic acid	297.2425−	C_18_H_34_O_3_	HMDB10736	0.90	<0.01 (↑)	0.13	−0.16
**XII**	Methylene disalicylate	287.0553−	C_15_H_10_O_6_		<0.01 (↑)	0.01 (↓)	0.01	−0.01
**XIII**	Lithocholic acid	375.2897−	C_24_H_40_O_3_	HMDB00761	0.01 (↓)	0.07	0.30	−0.14
**XIV**	*p*-Cresol (DC)	342.1154+	C_7_H_8_O	HMDB01858	0.18	0.04 (↓)	0.26	−0.07
**XV**	ND	325.1766+	C_16_H_24_N_2_O_5_		0.77	<0.01 (↓)	0.27	**0.18**
**XVI**	ND	226.1079+	C_11_H_15_NO_4_		0.21	<0.01 (↓)	0.05	0.13
**XVII**	Alanine (DC)	323.1056+	C_3_H_7_NO_2_	HMDB00161	0.85	0.01 (↓)	0.49	0.12
**XVIII**	Stercobilin	595.3514+	C_33_H_46_N_4_O_6_	HMDB0240259	0.97	0.02 (↓)	0.50	−0.02
**XIX** **XIX’**	Pentadecanoic acid Pentadecanoic acid (HQ)	241.2164− 384.3009+	C_15_H_30_O_2_	HMDB00826	0.09	<0.01 (↓)	0.07	−0.05
**XX** **XX’**	Dihydroxyquinoline	162.0552+ 160.0394−	C_9_H_7_NO_2_	HMDB04077	0.71	<0.01 (↓)	0.20	−0.02
**XXI**	2-Aminooctanoic acid	160.135+	C_8_H_17_NO_2_	HMDB00991	0.09	<0.01 (↓)	0.86	**0.20**
**XXII**	Hydroxy-hexadecenoic acid	271.2269−	C_16_H_32_O_3_	HMDB10734	0.03 (↓)	0.03 (↓)	0.17	−0.04
**XXIII**	Deoxycholic acid (HQ)	534.3691+	C_24_H_40_O_4_	HMDB00626	0.06	0.01 (↓)	0.56	0.10
**XXIV**	Phenylacetic acid	278.129+	C_8_H_8_O_2_	HMDB00209	0.47	<0.01 (↓)	0.69	−0.05
**XXV**	Heptadecanoic acid	269.2476−	C_17_H_34_O_2_	HMDB02259	0.15	<0.01 (↓)	0.22	−0.02

^1^ Pearson correlation coefficient between metabolite and ADG. Numbers in bold mean *p <* 0.05. ^2^ Derivatives were from two derivatization reactions using 2-hydrazinoquinoline (HQ) and dansyl chloride (DC). ^3^ (↑): Increased by fiber or AB; (↓): decreased by fiber or AB. ^4^ ND = Not determined.

## Data Availability

The data generated and analyzed in this study that support the conclusions are included within the article.

## Supplementary Materials

The following are available online at https://www.mdpi.com/article/10.3390/metabo12080686/s1. Table S1: sources of chemicals and reagents used in chemical analysis, LC-MS analysis, and structural confirmation and quantification; Table S2: ingredient and nutrient composition of experimental diets.
