# Supplementary material for: Identification of Independent and Shared Metabolic Responses to High-Fiber and Antibiotic Treatments in Fecal Metabolome of Grow–Finish Pigs
Source: Metabolites. 2022 Jul 26;12(8):686. doi: 10.3390/metabo12080686 (PMC9331191; doi:10.3390/metabo12080686)
Supplement: Supplementary file 1 [file metabolites-12-00686-s001.zip › metabolites-1825777-supplementary.pdf]

Identification of Independent and Shared Metabolic Responses to High-Fiber and Antibiotic Treatments in Fecal Metabolome of Grow–Finish Pigs

Yuan-Tai Hung [1,†], Yajian Song [2,†], Qiong Hu [3], Richard J. Faris [3], Juanjuan Guo [2], Yiwei Ma [2], Milena Saqui-Salces [1], Pedro E. Urriola [1,4], Gerald C. Shurson [1] and Chi Chen [1,2,*]

1 Department of Animal Science, University of Minnesota, 1988 Fitch Ave, Saint Paul, MN 55108, USA; hungx121@umn.edu (Y.-T.H.); msaquisa@umn.edu (M.S.-S.); urrio001@umn.edu (P.E.U.); shurs001@umn.edu (G.C.S.)
2 Department of Food Science and Nutrition, University of Minnesota, 1334 Eckles Ave, Saint Paul, MN 55108, USA; songyajian@tust.edu.cn (Y.S.); gjjfst15@163.com (J.G.); maxxx792@umn.edu (Y.M.)
3 Cargill Animal Nutrition, 10383 165th Ave NW, Elk River, MN 55330, USA; qiong_hu@cargill.com (Q.H.); richard_faris@cargill.com (R.J.F.)
4 Department of Veterinary Population Medicine, University of Minnesota, 1365 Gortner Ave, Saint Paul, MN 55108, USA
* Correspondence: chichen@umn.edu; Tel.: +1-612-624-7704; Fax: +1-612-625-5272
† These authors contributed equally to this work.

SUPPORTING INFORMATION

Table S1. Sources of chemicals and reagents used in chemical analysis, LC-MS analysis, structural confirmation and quantification.

**Chemicals and reagents**	**Vendor**
Taurocholic acid, Pentadecanoic acid	Acros Organics (Morris Plains, NJ, USA)
p-Chloro-L-phenylalanine	Alexis Biochemicals (San Diego, CA, USA)
Heptadecanoic acid, 2-Hydrazinoquinoline (HQ), Hyodeoxycholic acid, Triphenylphosphine (TPP)	Alfa Aesar (Ward Hill, MA, USA)
Methanol (LC-MS grade)	Avantor performance materials (Radnor, PA, USA)
Bacitracin	Alpharma Inc. (Bridgewater, NJ, USA)
Acetonitrile (LC-MS grade), Ammonium formate, Formic acid (LC-MS grade), Water (LC-MS grade)	Fisher Scientific (Houston, TX, USA)
p-Cresol	Fluka (Buchs, Switzerland)
2-2´-Dipyridyl disulfide (DPDS)	MP Biomedicals, LLC (Irvine, CA, USA)
Fatty acids standards (C4-C22)	Nu-Chek Prep, Inc. (Elysian, MN, USA)
Amino acid mixture (acidic), Amino acid mixture (basic), Dansyl chloride (DC), Deoxycholate, 3-Indoxyl sulfate potassium, Lithocholate, Phenylacetic acid, Sodium deoxycholate, Sodium taurochenodeoxycholate, Taurodeoxycholic acid sodium salt	Sigma-Aldrich (St. Louis, MO, USA)
Lauroylglycine	Santa Cruz Biotech (Santa Cruz, CA, USA)

Table S2. Ingredient and nutrient composition of experimental diets (as-fed basis) [1]

Fiber	Low				High			
Phase	1	2	3	4	1	2	3	4
Ingredients, %								
Corn [2]	61.58	64.90	63.27	67.72	49.93	45.51	40.61	36.98
Wheat middlings	10.00	10.00	15.00	15.00	25.00	35.00	45.00	55.00
Soybean meal	24.79	21.75	19.00	14.20	21.38	16.20	11.80	5.18
Poultry fat	1.03	0.71	0.75	0.87	1.03	0.71	0.75	0.87
Calcium carbonate	0.92	0.90	0.79	0.71	1.19	1.17	0.84	0.77
Monocalcium phosphate	0.71	0.58	0.38	0.43	0.56	0.33	0.19	0.18
Salt	0.43	0.43	0.59	0.42	0.43	0.43	0.59	0.42
Titanium dioxide	-	0.20	-	0.20	-	0.20	-	0.20
Sodium carbonate	-	-	-	0.02	-	-	-	0.00
Vitamin/Mineral premix [3]	0.11	0.11	0.11	0.11	0.11	0.11	0.11	0.11
DL-Methionine	0.06	0.05	-	0.01	0.04	0.02	-	0.00
L-lysine HCl	0.28	0.28	0.11	0.26	0.26	0.25	0.12	0.25
Threonine	0.09	0.09	0.00	0.06	0.08	0.07	0.00	0.06
Total	100	100	100	100	100	100	100	100
Calculated composition								
Dry matter, %	87.98	87.96	87.88	88.10	87.97	87.93	87.92	88.00
Net Energy, kcal/kg	2400	2400	2400	2440	2289	2219	2202	2169
Crude protein, %	18.72	17.54	16.39	14.94	18.71	17.60	16.39	15.00
Standardized ileal digestible Lys	1.07	1.00	0.80	0.80	1.02	0.92	0.74	0.71
Standardized ileal digestible Met	0.33	0.30	0.24	0.24	0.30	0.27	0.23	0.21
Standardized ileal digestible M+C	0.60	0.56	0.49	0.47	0.57	0.54	0.50	0.47
Standardized ileal digestible Thr	0.66	0.61	0.50	0.50	0.63	0.57	0.46	0.44
Crude fat, %	3.53	3.27	3.67	3.69	3.58	3.36	3.79	3.94
Crude ash, %	4.91	4.79	4.46	4.15	5.35	5.36	4.90	4.78
NDF, %	9.49	9.50	10.95	10.54	12.99	15.35	17.67	19.71
Total Ca, %	0.61	0.57	0.51	0.47	0.70	0.65	0.51	0.47
Digestible P, %	0.23	0.20	0.16	0.16	0.22	0.19	0.15	0.14

[1]In each phase of feeding, Bacitracin and fiber are two factors in the 2 × 2 factorial design of 4 diets, including Bacitracin free-low fiber (AF+LF) diet; Bacitracin free-high fiber (AF+HF) diet; Bacitracin-low fiber (AB+LF) diet; Bacitracin-high fiber (AB+HF) diet.

[2]Bacitracin (25 mg/kg) added at expense of corn

[3]Premix supplied the following nutrients per kilogram of diet: 60 IU Vitamin E Equivalent, 10,000 IU Vitamin A, 1,600 Vitamin D3 Equivalent, 1.5 mg Thiamine, 8 mg Riboflavin, 50 mg Niacin, 60 mg Pantothenic Acid, 2 mg Pyridoxine, 1.1 mg Folic Acid, 35 µg Vitamin B12, 3.5 mg Menadione, 75 µg Ethoxyquin, 18 mg Copper, 110 mg Iron, 0.7 mg Iodine, 50 mg Manganese, 110 mg Zinc, 0.3 mg Selenium
